# Antiproliferative, Apoptotic, and Autophagic Activity of Ranibizumab, Bevacizumab, Pegaptanib, and Aflibercept on Fibroblasts: Implication for Choroidal Neovascularization

**DOI:** 10.1155/2015/934963

**Published:** 2015-09-27

**Authors:** Lyubomyr Lytvynchuk, Andrii Sergienko, Galina Lavrenchuk, Goran Petrovski

**Affiliations:** ^1^Professor Sergienko Eye Clinic, 47 A Pirogova Street, 21008 Vinnycia, Ukraine; ^2^Department of Ophthalmology, Faculty of Medicine, Albert Szent-Györgyi Clinical Center, University of Szeged, Korányi fasor Ulica 10-11, 6720 Szeged, Hungary; ^3^Stem Cells and Eye Research Laboratory, Department of Biochemistry and Molecular Biology, Faculty of Medicine, University of Debrecen, Egyetem Téren 1, 4032 Debrecen, Hungary; ^4^State Institution “Research Center for Radiation Medicine of Academy of Medical Sciences of Ukraine”, Laboratory of Cell Radiobiology, 53 Melnykova Street, 04050 Kyiv, Ukraine

## Abstract

*Purpose*. Choroidal neovascularization (CNV) is one of the most common complications of retinal diseases accompanied by elevated secretion of vascular endothelial growth factor (VEGF). Intravitreal anti-VEGFs (ranibizumab, bevacizumab, pegaptanib, and aflibercept) can suppress neovascularization, decrease vascular permeability and CNV size, and, thereby, improve visual function. The antiproliferative, apoptotic, and autophagic effect of anti-VEGF drugs on fibroblasts found in CNVs has not been yet explored. *Methods*. Concentration-dependent cellular effects of the four anti-VEGFs were examined in L929 fibroblasts over a 5-day period. The cell survival, mitotic and polykaryocytic indices, the level of apoptosis and autophagy, and the cellular growth kinetics were all assessed. *Results*. The anti-VEGFs could inhibit the survival, mitotic activity, and proliferation as well as increase the cellular heterogeneity, apoptosis, and autophagy of the fibroblasts in a dose-dependent manner. Cellular growth kinetics showed ranibizumab to be less aggressive, but three other anti-VEGFs showed higher antiproliferative and apoptotic activity and expressed negative cellular growth kinetics. *Conclusions*. The antiproliferative, apoptotic, and autophagic activity of anti-VEGFs upon fibroblasts may explain the cellular response and the etiology of CNV involution *in vivo* and serve as a good study model for CNV *in vitro*.

## 1. Introduction

**Figure 1:**
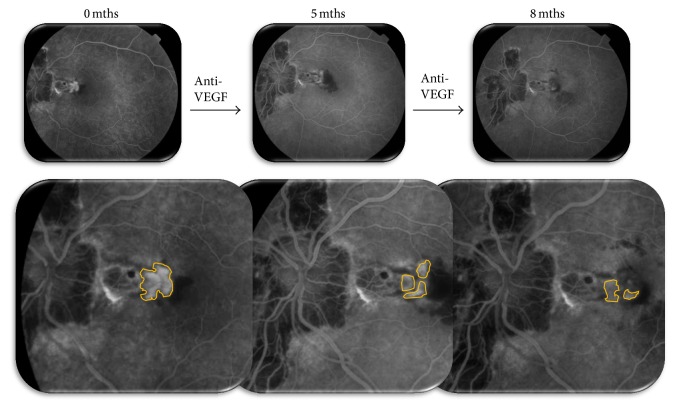
Choroidal neovascularization (CNV) dynamics after repeated anti-VEGF therapy (CNV size is circumscribed with yellow color; images shown are at the 40th second of fluorescein angiography).

The development of choroidal neovascularization (CNV) is one of the most sight threatening complications of different retinal diseases such as age-related macular degeneration, pathologic myopia, angioid streaks, and choroidal rupture. Foveal or extrafoveal location of CNV limits the use of lasers due to its potential side effects on the surrounding healthy tissue. The effectiveness of intravitreal administration of different antivascular endothelial growth factors (anti-VEGFs) is well known in the treatment of CNV of different origin [1–4]. The mechanism how intravitreal injections (IVIs) of such drugs work is complex and involves blocking of various types of VEGFs, decreased permeability of newly formed blood vessel walls, and reduced swelling of the retinal layers. Our optical coherence tomography (OCT) and fluorescein angiography (FA) performed before and after IVI of anti-VEGF drugs have revealed significant involution and decrease of CNV size (Figure 1).

The exact mechanism which leads to decrease of the CNV dimensions is not well understood. During recent years, a number of studies have published the impact of anti-VEGF drugs upon different cellular cultures* in vitro* [5–10]. Fibroblasts and myofibroblasts being among the most common cells found within the cellular matrix of CNVs and known to have high mitotic activity [7] have not been examined for their cellular effects upon anti-VEGF drug treatment. Our goal was to investigate the antiproliferative, apoptotic, and autophagic effects of anti-VEGF drugs on a fibroblast-like cell strain which can serve as* in vitro* model for CNV cellular matrix formation and to analyze the dose dependence regarding antiproliferative activity.

## 2. Materials and Methods

### 2.1. Cell Culture and Treatment Regimes

*In vitro* studies were performed using a fibroblast-like mouse cell strain L929 obtained from ATCC and cultivated according to conventional methods [11, 12] and nutrient medium composed of RPMI-1640 supplemented with fetal calf serum (10%) and gentamicin (10 mg/mL). Cultivation of the cell strain with different concentration of anti-VEGF drugs was performed as described below.

Ranibizumab (Lucentis, Novartis, Switzerland), a fragment of a human monoclonal antibody against VEGF-A, which is secreted by recombinant strain of* Escherichia coli* and its isoforms selectively bind to VEGF-A (VEGF_110_, VEGF_121_, and VEGF_165_), was added to the culture 24 hours after fresh cell plating in concentrations of 12.5, 50, 125, and 250 *μ*g/mL.

Bevacizumab (Avastin, Genetech/Roche, USA), a monoclonal antibody against VEGF, which is used off-label to treat various eye diseases in which increased concentration of VEGF is found and neovascularization is present, was added to the culture 24 hours after fresh cell plating at concentrations of 0.65, 3.13, 6.5, and 12.5 *μ*g/mL.

Pegaptanib (Macugen, Pfizer, USA), a pegylated modified oligonucleotide that binds selectively and with high affinity to an extracellular VEGF_165_, was added to the culture 24 hours after fresh cell plating at concentrations of 0.075, 0.15, 0.3, 0.75, and 1.5 *μ*g/mL.

Aflibercept (Eylea, Bayer HealthCare, Germany), a fusion protein approved in the United States and Europe for the treatment of wet form of age-related macular degeneration, working by binding to circulating VEGF (subtypes VEGF-A and VEGF-B), as well as to placental growth factor (PGF), thus inhibiting growth of new blood vessels in the choriocapillaris [2], was added to the culture 24 hours after cell plating at concentrations of 0.04, 0.08, 0.2, 0.4, and 0.5 *μ*g/mL.

Minimal drug concentrations were established according to the appearance of a multiplex of cellular effects (cellular proliferation, mitotic activity, polykaryocytic index, and apoptosis) and applied into the study, while maximal concentrations were determined with relevance and close approximation to the ones used in clinical practice (e.g., 0.5 mg/4 mL vitreous volume for ranibizumab; 1.25 mg/4 mL vitreous volume for bevacizumab; 0.3 mg/4 mL vitreous volume for pegaptanib; 2.0 mg/4 mL vitreous volume for aflibercept; all of the anti-VEGFs are used clinically at 1–3-month interval).

### 2.2. Cellular Vital Parameters

Different cellular responses were evaluated on a daily basis up to 5 days. The following cellular vital activity indices were evaluated: cellular growth/expansion and mitotic and polykaryocytic indices (PKI). For cultivation, 5 × 10^4^ cells were added to the cell culture dishes covered by culture glass slides (size 16 × 8 mm) and filled up to 1 mL of medium and then left to form monolayers within 5 days. Anti-VEGFs were added to the cultures 24 hours after cell plating in different concentrations specified accordingly. The samples were fixed for analysis in 96% ethanol and then stained by hematoxylin and eosin (H&E). The total number of cells and the count of mitoses and polykaryocytes (2 or more nuclei) were determined under optical microscope (Axioscope, Germany) at 1000x magnification within a grid area of 0.05 mm^2^. Mitotic index and PKI were adjusted to 1000 cells (‰). Parallely, cellular vital indices were evaluated within the intact cellular culture.

### 2.3. Detection of Apoptosis and Autophagy

The level of apoptosis was determined on the same cultures in which the cellular vital indices were analyzed. The cells were first washed in phosphate buffered saline (PBS) and then detached for 10 minutes in trypsin and suspended again in PBS. Consequently, centrifugation (1400 rpm, 5 min) and resuspension of the cells in propidium iodide (1 *μ*g/mL) were performed. The level of apoptosis was determined according to the number of apoptotic cells in pre-G1 phase using ductal cytofluorimeter FACStar Plus (Becton Dickinson, USA).

Anti-LC3 polyclonal antibody was purchased from Novus Biologicals, USA (NB100-2220-0.1) for analysis of autophagy. Cell lysates were prepared from each condition after which equal amounts of protein were loaded onto the gel. Proteins were separated on a NuPAGE 15% Bis-Tris polyacrylamide gel and then transferred onto Immobilon-P Transfer Membrane (Millipore, IPVH00010). Membranes were blocked in Tris buffered saline containing 0.05% Tween-20 (TBS-T) and 5% nonfat dry milk (BioRad, 170-6435, 170-6531, and 100-04504-MSDS) for 1 hour. After blocking, membranes were probed overnight at 4°C with the anti-LC3 antibody in dilution buffer (TBS-T containing 1% nonfat dry milk), followed by 1-hour incubation with a peroxidase-conjugated rat anti-rabbit secondary antibody (Sigma, A6154) for 1 hour at room temperature. Peroxidase activity was detected with SuperSignal West Femto Maximum Sensitivity Chemiluminescent Substrate (Pierce, 34095) using a Lumi-Imager (Roche Diagnostics, Mannheim, Germany).

### 2.4. Cellular Growth Kinetics

Proliferative activity of cells was determined according to their growth kinetics parameters: specific growth velocity (*μ*), population doubling time (*t*_*d*_), and reproduction velocity (*n*) at Day 5 of the observation [13]. The specific growth velocity of the culture in phase of logarithmic growth was calculated using the formula *μ* = (ln⁡*X* − ln⁡*X*_*o*_)*t* ^−1^, where *X* is the cell quantity after certain time interval *t* (Day 5 of cultivation), *X*_*o*_ is the cell quantity on Day 1 of cultivation, and *t* is the time of observation (5 days of cultivation). Using specific growth velocity, population doubling time was calculated as *t*_*d*_ = ln⁡2/*μ* = 0693*μ*. Reproduction velocity (*n*) was determined using the formula *n* = 3.32log⁡⁡(*X*/*X*_*o*_).

### 2.5. Statistical Analysis

Results were statistically analyzed by Student's *t*-test using Microsoft Excel and Biostat (Primer of Biostatistics, Version 4.03, by Stanton A. Glantz). *P* < 0.05 was considered significant. If not otherwise noted, all the experiments were performed three times independently.

## 3. Results

### 3.1. Morphological and Functional Characteristics of L929 Cells Treated by Anti-VEGFs

**Figure 2:**
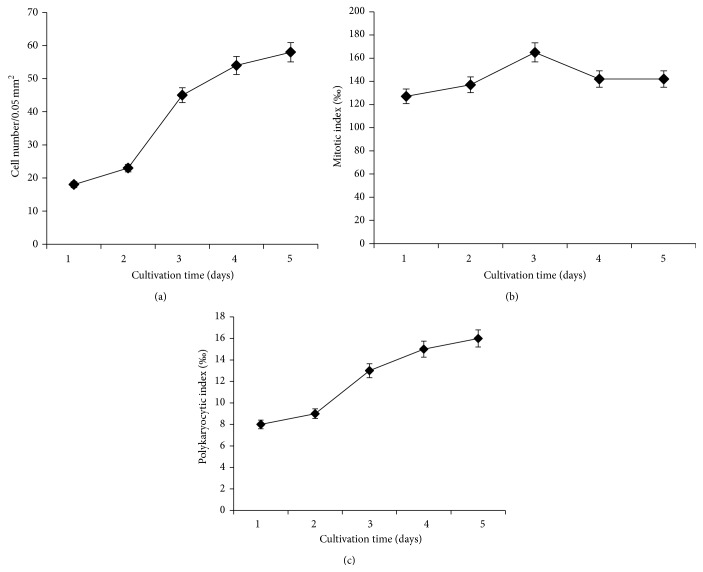
Kinetics of the cellular survival (a) and mitotic (b) and polykaryocytic indices (c) in untreated, control L929 cells.

Under normal conditions, L929 cells form a dense cellular monolayer with the majority of the cells acquiring polygonal and spindle shape morphology. The cells have relatively large nuclei, light cytoplasmic vacuoles, and small granules with occasional di- and trinucleated cells found in the culture. On average, 2 to 5 cells at different stages of mitosis can be detected per visual field in the untreated culture, assuming round shape, small cytoplasm, and hyperchromatic nuclei due to condensation of the chromatin under mitosis. The intact cells have a characteristic proliferative activity that increases from Day 1 to Day 5 of cultivation (logarithmic growth phase), reaching growth plateau at Day 6 (stationary growth phase), when the density of the cellular monolayer becomes highest (58.0 ± 2.7/0.05 mm^2^) (Figure 2(a)). Maximum mitotic activity is observed at Day 3 of cultivation (166.0 ± 7.5‰), with a decrease in the mitotic index at Day 4 due to contact inhibition and confluency of the cellular culture. The PKI in the intact control cells varies between 8 and 12‰.

**Figure 3:**
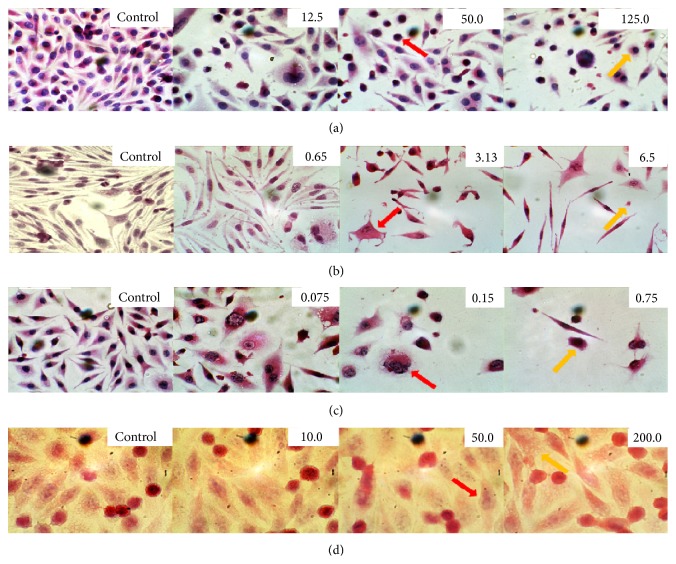
Cellular effects of ranibizumab (a), bevacizumab (b), pegaptanib (c), and aflibercept (d) on L929 cells. Cells shown are at Day 5 of various treatment concentrations (H&E staining, magnification ×1000).

**Figure 4:**
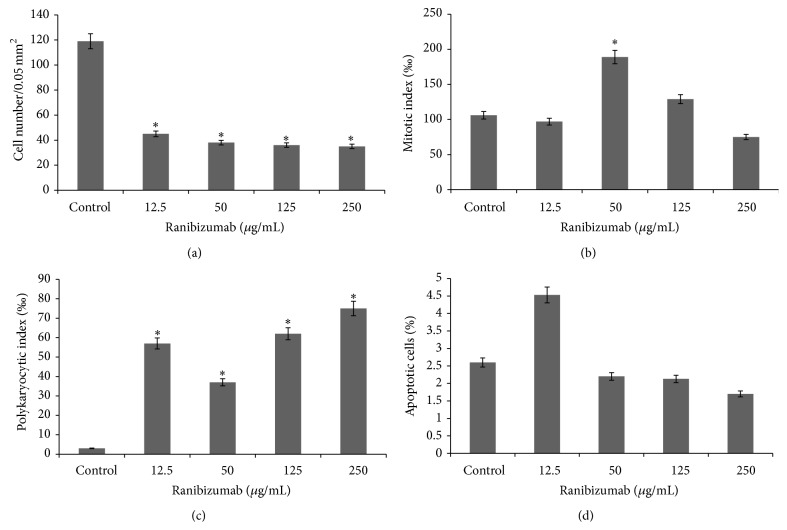
Kinetics of cellular proliferation (a), mitotic activity (b), polykaryocytic index (c), and apoptosis (d) in L929 cells treated by ranibizumab at different concentrations (data shown are at Day 5 of the treatment; *n* = 3, ^∗^ *P* < 0.05).

Incubation of the L929 cells with ranibizumab at a concentration of 12.5 *μ*g/mL leads to decreased density of the culture monolayer by 3 times (Figures 3(a) and 4(a)), which is caused by the appearance of increased number of cells with apoptotic features—decreased cytoplasm and condensed chromatin in the nucleus (Figures 3(a) (red arrow) and 4(d)). The treated cells assume mainly spindle-shape morphology, while their PKI increases by 54‰ (Figure 4(c)), a sign of cell demise. At higher concentrations up to 125.0 *μ*g/mL, the heterogeneity of the cell culture increases, with a predominant cellular morphology being round and polygonal (Figure 3(a) (yellow arrow)). The number of polykaryocytes markedly increases to 62‰, while the mitotic index remains relatively stable compared to the control (Figure 4(b)). Incubation of the L929 cells at a dose of 250 *μ*g/mL results in severe degradation of the cellular structure and strong vacuolization of the cytoplasm.

**Figure 5:**
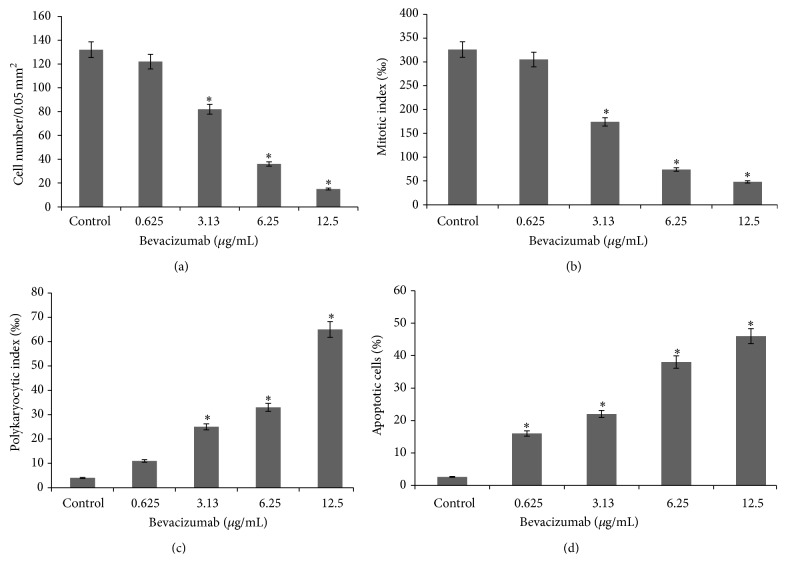
Kinetics of cellular proliferation (a), mitotic activity (b), polykaryocytic index (c), and apoptosis (d) in L929 cells treated by bevacizumab at different concentrations (data shown are at Day 5 of the treatment; *n* = 3, ^∗^ *P* < 0.05).

Exposure to bevacizumab at concentration of 0.65 *μ*g/mL induces no morphological changes in the L929 cells compared to the control (Figure 3(b)). Increasing the dose up to 3.13 *μ*g/mL leads to an increase in the heterogeneity of cellular culture, with round and polygonal cellular morphology becoming more prominent (Figure 3(b) (red arrow)). A small number of mitotic cells with vacuolar cytoplasm can be observed compared to the control, while the number of polykaryocytes increases 6.3 times (Figure 5(c)), and the mitotic index and cell number decrease by 1.9 and 1.6 times, respectively, compared to the control (Figures 5(a) and 5(b)). At higher concentrations of bevacizumab (6.25 and 12.5 *μ*g/mL), a severe degradation of cellular structure, with the cytoplasm becoming full of vacuoles, and a large number of apoptotic cells appear (38.0 ± 1.5 and 46.0 ± 2.2, resp.) (Figures 3(b) (yellow arrow) and 4(d)). A significant reduction in the cell density and mitotic activity as well as increase in the PKI is noticed at concentrations higher than 0.625 *μ*g/mL (*P* < 0.05) (Figure 5(c)).

**Figure 6:**
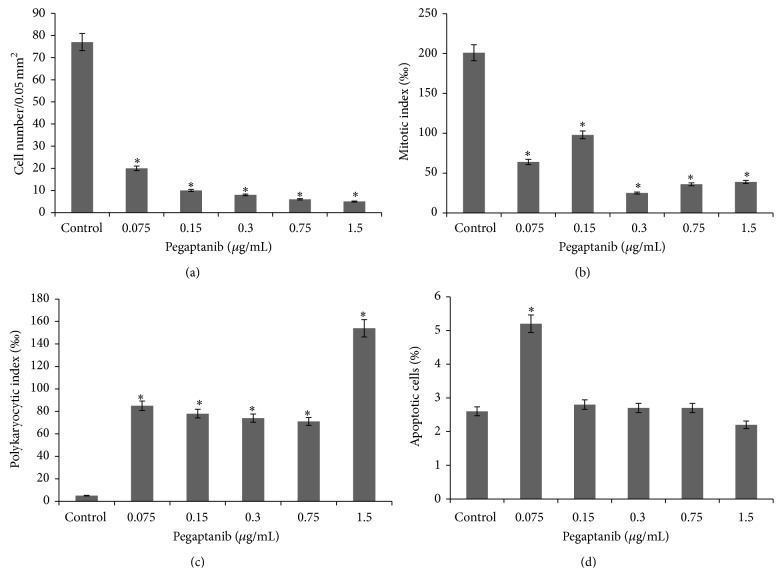
Kinetics of cellular proliferation (a), mitotic activity (b), polykaryocytic index (c), and apoptosis (d) in L929 cells treated by pegaptanib at different concentrations (data shown are at Day 5 of the treatment; *n* = 3, ^∗^ *P* < 0.05).

Incubation of cells with pegaptanib shows pronounced antiproliferative effects of the drug from its lowest dose (0.075 *μ*g/mL) (Figure 6(a)) and an almost doubling of the apoptotic cells in the culture. This is manifested by a significant decrease in the cell number and mitotic activity compared to the controls (*P* < 0.05) (Figures 6(a) and 6(b)). Increasing the dose up to 1.5 *μ*g/mL increases the antiproliferative effect which is manifested through a reduction in the density of the cells and the appearance of vacuoles in the cytoplasm (Figure 3(c) (red arrow)). Cells with signs of apoptosis also appear at higher concentrations (Figure 3(c) (yellow arrow)), but their number, paradoxically, is similar to that of the control cells (Figure 6(d)), while the number of mitoses and presence of polykaryocytes decreases and increases, respectively (Figures 6(b) and 6(c)).

**Figure 7:**
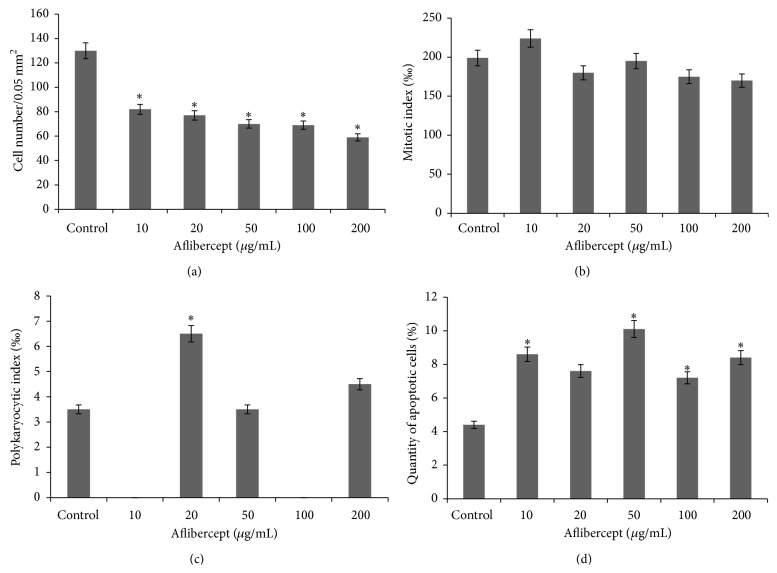
Kinetics of cellular proliferation (a), mitotic activity (b), polykaryocytic index (c), and apoptosis (d) in L929 cells treated by aflibercept at different concentrations (data shown are at Day 5 of the treatment; *n* = 3  ^∗^ *P* < 0.05).

Exposure to aflibercept causes not much morphological difference in the L929 cells compared to the control; for example, the cells remain predominantly polygonal and spindle shaped with centrally situated well-colored round or oval nuclei (Figure 3(d) (red arrow)). The cytoplasm has mesh structure and becomes slightly vacuolized under aflibercept treatment (Figure 3(d) (yellow arrow)), with many cells appearing at different stages of mitosis and, under certain concentrations, an entire absence of polykaryocytes' formation is seen compared to control (Figure 7(c)). The cell number is reduced by at least 1.6-fold with drug concentration of 10 *μ*g/mL and by 2.2-fold when exposed to a maximum concentration of 200 *μ*g/mL (Figure 7(a)). Interestingly, the mitotic index does not change compared to the control, which corresponds well to the morphological cellular stability observed (Figure 7(b)). Furthermore, complete lack of polykaryocytes is observed under certain concentrations (10 and 100 *μ*g/mL) (Figure 7(c)). There is no statistical difference between PKI of control cells and under 50 or 200 *μ*g/mL treatment with aflibercept (*P* < 0.05). The number of apoptotic cells (Figure 7(d)) increases and depends in a nonlinear manner on the drug concentration.

### 3.2. Cellular Growth Kinetics of L929 Cells under Different Treatments

**Figure 8:**
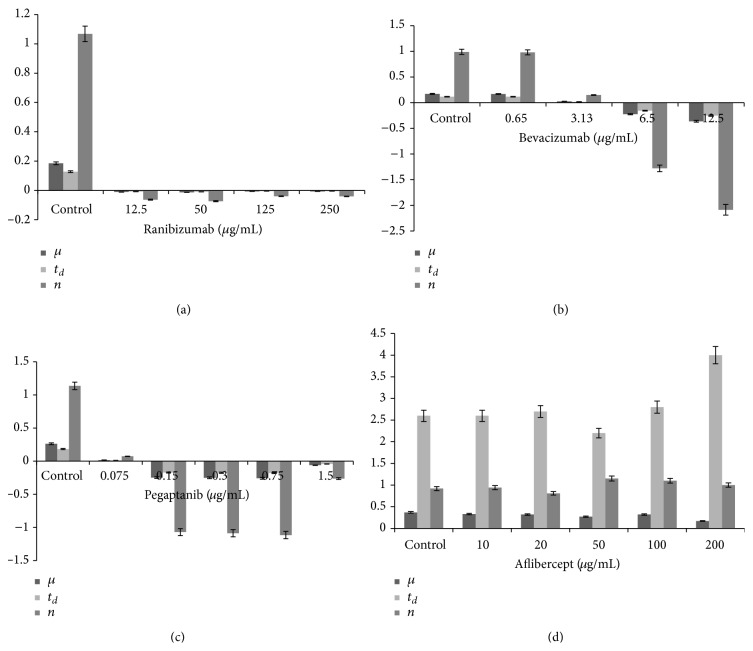
Comparison of the cellular growth kinetics in L929. Specific growth velocity (*μ*), population doubling time (*t*_*d*_), and cell reproduction velocity (*n*) are shown under treatment with ranibizumab (a), bevacizumab (b), pegaptanib (c), and aflibercept (d) at different concentrations (data shown are at Day 5 of the treatment; *n* = 3).

Comparison of the cellular growth effect of different concentrations of anti-VEGF drugs is shown in Figure 8. According to the cellular growth kinetic parameters (specific growth velocity (*μ*), population doubling time (*t*_*d*_), and cell reproduction velocity (*n*)), ranibizumab seems to be less aggressive compared to the rest of the anti-VEGFs studied, the cellular death being compensated by cellular reproduction in its case (Figure 8(a)) [13]. Minimal concentrations of bevacizumab cause faster cellular proliferation than death (Figure 8(b)); however, this turns around under higher concentrations, when generalized cell death through initiation of apoptosis occurs. The action of pegaptanib appears to be antiproliferative at lowest concentrations, while the balance between survival, reproductive activity, and death is shifted towards death at higher concentrations, meaning that the destruction in cellular culture runs faster than cellular mitosis (Figure 8(c)). Treatment by aflibercept (Figure 8(d)) causes statistically significant reduction in the cell number, in particular, concentrations of 50 to 200 *μ*g/mL, while, at the same time, the population doubling time decreases (at 50 *μ*g/mL), and the rate of cell proliferation increases. This indicates a shift of the balance of cellular growth and death in the culture towards death, and, according to the determination of number of apoptotic cells (Figure 8(d)), this death is mainly caused by apoptosis. Concentration of 200 *μ*g/mL slows down the progression of cellular growth, which indicates linking of the reproductive death (pathologic mitosis) to apoptosis.

### 3.3. Induction of Autophagy by Ranibizumab and Bevacizumab in L929 Cells

**Figure 9:**
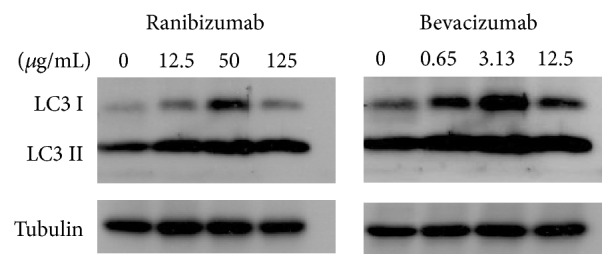
Induction of autophagy by ranibizumab and bevacizumab in L929 cells.

The appearance of vacuoles in the cytoplasm of L929 cells under different treatments by ranibizumab and bevacizumab was further examined for the presence of autophagy. Both treatment modalities induce conversion of the cytoplasmic form of the myosin light chain kinase 3 (LC3) I into the autophagic vacuoles-bound LC3 II, with the highest concentrations of each drug inducing highest conversion of LC3 I to LC3 II (quantification data not shown) (Figure 9).

## 4. Discussion

Over the past decade, IVIs of anti-VEGF drugs take the leading place among the treatment modalities used for retinal diseases with increased production of VEGF [1–4]. However, only a few studies are dedicated to the side effects these drugs have on ocular tissues being exposed [5–10]. To our knowledge, this is the first study which compares antiproliferative action of ranibizumab, bevacizumab, pegaptanib, and aflibercept on fibroblast-like cells* in vitro *to elucidate the different dose-dependent properties and implications for CNV.

Our data show that all four anti-VEGFs demonstrate antiproliferative activity on the L929 cells over a 5-day study period. Starting from the lowest concentrations used, the heterogeneity of the cellular monolayer increases as a result of depression of mitosis and survival, while the number of apoptotic cells increases. Increasing the concentrations of each of the four anti-VEGFs results in exacerbation of the above-mentioned effects.

The growth kinetic analysis reveals concentration-dependent antiproliferative and apoptotic effects of all anti-VEGFs, except for ranibizumab, where higher cellular reproduction occurs with concentration increase, and, therefore, the concentration-dependent cellular growth is partially compensated by reproduction. The ranibizumab proves, therefore, to be less aggressive than other anti-VEGFs in regard to its antiproliferative activity.

A recent study compared the antiproliferative and cytotoxic effects of bevacizumab, pegaptanib, and ranibizumab on different ocular cells, except fibroblasts [8]. Ranibizumab reduced the cell proliferation by 44.1%, while bevacizumab and pegaptanib reduced it by 38.2% and 35.1%, respectively, when applied to choroidal epithelial cells (CECs), although the difference was not found to be statistically significant. A slight antiproliferative effect of bevacizumab and pegaptanib was also revealed on adult retinal pigment epithelium (ARPE19 cell line). Ranibizumab neither had the same effect on cell proliferation of ARPE19 cells nor did it have cytotoxicity on retinal ganglion cells (RGC5), CECs, and ARPE19 cells. It could also efficiently block migration, but not proliferation induced by growth factor combinations, including VEGF in retinal endothelial cells.

Another study collated the effects of ranibizumab, pegaptanib, and bevacizumab on the different stages of angiogenesis using cultivation of drugs on human umbilical vein endothelial cells (HUVEC) [5]. According to the results, apoptosis of HUVEC was markedly increased by ranibizumab and bevacizumab. Clinically used doses of these drugs, but not pegaptanib, caused significantly reduced cellular proliferation without causing cytotoxic effects at all concentrations used. Finally, incubation of HUVEC with anti-VEGF drugs caused a decreased expression of the active form of the VEGF receptor-2, with bevacizumab causing 66% of control and ranibizumab and pegaptanib causing 86% decrease compared to the control.

A separate study compared the cytotoxicity and antiproliferative activity of aflibercept, bevacizumab, and ranibizumab on different ocular cells (ARPE19, RGC-5, and 661W) [7] and concluded that aflibercept does not affect cellular viability or induce apoptosis. Albeit aflibercept had slight upregulation and downregulation effects on certain VEGF-related factors, however, those were not significant when compared to bevacizumab and ranibizumab.

Our experimental study explored the cellular effects of four different anti-VEGFs on L929 cells as a model of the fibroblast-based cellular matrix of CNV* in vitro*. The results revealed their effect on the proliferative activity (survival, proliferative and mitotic activity, and apoptosis) and their hormesis; that is, small doses of the drugs (ranibizumab 12.5 *μ*g/mL, bevacizumab 3.13 *μ*g/mL, pegaptanib 0.15 *μ*g/mL, and aflibercept 0.04 *μ*g/mL) exhibited a pronounced antiproliferative effect on the cellular culture, while bevacizumab in all concentrations increased apoptosis of the L929 cells. Inhibition of the proliferation and increased heterogeneity of these cells under anti-VEGF treatment are a sign of reproductive cellular death.

When cultured with bevacizumab, pegaptanib, and aflibercept, the L929 cells showed marked dose-dependent effects which were manifested by an increase in the antiproliferative action with increasing dose. Inversely, ranibizumab caused compensation of the antiproliferative and apoptotic action by cellular proliferation in spite of increasing drug concentration. This compensation can probably be partially due to autophagy, which is a self-digestive or self-recycling mechanism found in cells.

The reasons why L929 cell strain was chosen in this study were the absence of background VEGF secretion, as well as exploring the alternative cellular effects of anti-VEGF drugs as an* in vitro* model for CNV. Indeed, the most highly proliferating cell types amid the cellular types in CNV are fibroblasts and myofibroblasts [14]. L929 cells have also been used as* in vitro* model for vital cell, allowing to judge cellular effects of different drugs upon vital, highly proliferating cells.

Although the data regarding presence of absence of VEGF receptors on the cellular membrane of fibroblast-like cells in CNV is missing, other studies have shown VEGF receptor presence on joint fibroblasts extracted from humans as well as expression of VEGF by inflammatory stimulation of fibroblast-like cells that infiltrate the joint in a collagen-induced arthritis [15]. An obvious limitation of this study is the resemblance of the* in vitro* findings to clinical conditions, for example, CNV.

Antiproliferative and apoptotic properties of anti-VEGF drugs on fibroblast-like cells can explain an alternative, beneficial mechanism of their action on such cells and myofibroblasts found in CNV [14]. Inhibition of the cellular survival and mitosis by ranibizumab, bevacizumab, pegaptanib, and aflibercept in different concentrations has to be taken into consideration while using them in patients. Only ranibizumab, amid all anti-VEGFs studied, exhibits the slightest antiproliferative activity which allows for compensation of apoptosis by increased proliferation. The complete absence of polykaryocytes revealed after cultivation with aflibercept at concentrations of 10 and 100 *μ*g/mL may indicate the influence of the drug on the signal transfer from the membrane to the nucleus.

The identified properties of these drugs require further investigation of their action* in vitro* and* in vivo*. Additionally, further research pertaining to hormesis of anti-VEGFs needs to be performed to eliminate possible side effects on healthy retinal tissues.
